# County-wide assessments of Illinois white-tailed deer (*Odocoileus virginianus*) prion protein gene variation using improved primers and potential implications for management

**DOI:** 10.1371/journal.pone.0274640

**Published:** 2022-11-30

**Authors:** Daniel B. Raudabaugh, Yasuko Ishida, Nicholas J. Haley, William M. Brown, Jan Novakofski, Alfred L. Roca, Nohra E. Mateus-Pinilla

**Affiliations:** 1 Illinois Natural History Survey, University of Illinois, Champaign, Illinois, United States of America; 2 Department of Animal Science, University of Illinois, Urbana, Illinois, United States of America; 3 Department of Microbiology and Immunology, Midwestern University, Glendale, AZ, United States of America; 4 Department of Pathobiology, College of Veterinary Medicine, University of Illinois, Urbana, Illinois, United States of America; 5 Carl R. Woese Institute for Genomic Biology, University of Illinois at Urbana-Champaign, Urbana, Illinois, United States of America; US Geological Survey, UNITED STATES

## Abstract

Chronic wasting disease (CWD) is a fatal, highly infectious prion disease that affects captive and wild cervids. Chronic wasting disease is the only known transmissible spongiform encephalopathy affecting free-ranging wildlife. In CWD-positive deer, some haplotypes of the prion protein gene *PRNP* are detected at lower frequencies as compared to CWD-negative deer, as are some variants of the prion protein PrP. Here, we examined wild, hunter-harvested CWD-negative white-tailed deer (*Odocoileus virginianus*) to determine whether there were geographical or temporal differences in the *PRNP* haplotypes, *PRNP* diplotypes, PrP proteoforms, and in the proportion of deer with at least one protective haplotype. We sampled 96–100 hunter-harvested deer per county at two time points in the Illinois counties of Jo Daviess, LaSalle, and Winnebago, chosen based on their geographic locations and known occurrence of CWD. The entire coding region of *PRNP* was sequenced, with haplotypes, diplotypes, and PrP proteoforms inferred. Across time, in Winnebago there was a significant increase in PrP proteoform F (*p* = 0.034), which is associated with a lower vulnerability to CWD. In every county, there was an increase over time in the frequency of deer carrying at least one protective haplotype to CWD, with a significant increase (*p* = 0.02) in the Jo Daviess County CWD infected region. We also found that primer combination was important as there was an 18.7% difference in the number of the deer identified as homozygous depending on primer usage. Current Illinois state management practices continue to remove CWD infected deer from locally infected areas helping to keep CWD prevalence low. Nonetheless, continued research on spatial and temporal changes in *PRNP* haplotypes, PrP proteoforms, and levels of deer vulnerability among Illinois deer will be important for the management of CWD within the state of Illinois and beyond.

## Introduction

Chronic wasting disease (CWD) is an emerging 100% fatal disease known to infect both captive and wild members of the deer family Cervidae. The first clinical case of CWD was identified in a captive mule deer (*Odocoileus hemionus* Rafinesque) from the state of Colorado in the United States of America (USA) [1], and the first free-range clinical case was identified in an elk (*Cervus canadensis* Erxleben) [2] in Colorado. Chronic wasting disease is highly infectious, and CWD is the only transmissible spongiform encephalopathy known to affect wild free-ranging wildlife [3, 4]. Since its initial discovery, CWD has spread to at least half of the states within the USA, parts of Canada, Finland, Norway, Sweden, and South Korea [5, 6]. Management strategies have been enacted to prevent the spread of CWD, but CWD continues to spread geographically due to many factors including the ease of horizontal transmission from one individual to another [7] and the long-term environment persistence of prion proteins [8].

Cellular prion proteins (PrP^C^) are membrane-bound proteins produced by all mammals. In their normal conformation, they are degraded through conventional proteolytic pathways. However, PrP^C^ becomes infectious (PrP^Sc^) due to a post-translational misfold which prevents PrP^Sc^ from being degraded by cellular proteases [9]. The misfolded infectious protein is known as “prion” for proteinaceous infectious particle [9]. In addition, the PrP^Sc^ goes on to serve as a template for converting PrP^C^ to PrP^Sc^ [9]. Disease occurs because PrP^Sc^ (PrP^CWD^ for CWD) is insoluble, precipitates, and accumulates around neurons in the brain, where the prion protein gene (*PRNP*) is predominantly expressed. The accumulation causes neurons to die which leads to altered cognitive and behavioral function [10]. In captivity, white-tailed deer (*Odocoileus virginianus*) typically survive 16 to 30 months after contracting PrP^CWD^ [11]. White-tailed deer expressing clinical symptoms may survive up to one year, but most individuals succumb within three to four months [12]; however, the incubation period can be affected by the *PRNP* genotype [13].

In addition, CWD is influenced by genetic variation within the *PRNP* coding region. Non-synonymous polymorphisms in *PRNP* gene sequences have different frequencies between CWD positive and CWD negative white-tailed deer, suggesting a protective effect for some protein-altering mutations [14, 15]. A general overview of these variants across the family Cervidae is discussed by [16–18], [17] identified *PRNP* haplotype variants, and [18] inferred PrP proteoforms (protein variations encoded by a specific gene [19]) in white-tailed deer. Proteoform (PrP variant) A is overrepresented in CWD-positive individuals, while proteoforms C and F are underrepresented in CWD-positive individuals. Other researchers suggested that the SNP H95-PrP^C^ (which proteoform F carries) limits the peripheral accumulation of PrP^CWD^ based on immunohistochemistry [20]. More recent research [21] suggested that in a PrP homozygous background (two copies of the same protein variant (proteoform), the SNP S96-PrP^C^ may prevent stable propagation of the most common *PrP*^*CWD*^ *in vitro* when using the protein misfolding cyclic amplification diagnostic technique. These findings provide additional support that certain PrP proteoforms lower the vulnerability to CWD.

The genetic variability within a population may influence its vulnerability to CWD [22]. Consequently, past research has shown that *PRNP* sequencing help inform population-level vulnerability patterns [23] to potentially assist with the prediction of disease spread. Previous studies [22, 24] have focused on characterizing and evaluating the variations within the *PRNP* gene [22, 24]. Interestingly, a recent study showed that white-tail deer populations within the Mid-Atlantic states have *PRNP* genotypic variation at a fine geographic scale (sub-region and county level) [25], while little evidence for *PRNP* genotype geographic structure was found in the state of Nebraska, USA [26]. Unfortunately, limited research has been conducted on *PRNP* and PrP population frequencies at the county level (geographic scale) in other CWD-positive USA regions.

In the case of sequencing *PRNP* in white-tailed deer, evidence of allelic dropout has recently been reported using one of the more commonly used primer sets. Researchers identified a polymorphism in the forward primer binding site [14] that prevents primer annealing leading to allelic dropout [27]. Ultimately, this leads to incorrect *PRNP* genotyping for deer carrying the primer binding site polymorphism [27]. Therefore, it is important to determine the extent of this polymorphism in the Illinois wild white-tailed deer population in order to determine its effect on the accuracy of previous Illinois *PRNP* published frequencies.

Here, we examined populations of wild white-tailed deer in three Illinois counties (Jo Daviess, LaSalle, and Winnebago) to determine whether there are differences in composition and frequencies of *PRNP* haplotypes, *PRNP* diplotypes, inferred PrP proteoforms, and the number of deer that carry less vulnerable PrP proteoforms. We evaluated geographic differences between counties and temporal differences within counties. We expected to find temporal and geographic differences in composition and frequencies of *PRNP* haplotypes, *PRNP* diplotypes, PrP proteoforms, and PrP proteotypes. Diplotype is defined as the two haplotypes within each individual diploid deer, and PrP proteotype is defined as the two proteoforms encoded by each haplotype within each individual. We evaluated the *PRNP* allelic frequencies obtained using primers by [14] (O’Rourke primers) and by [27] (Haley primers) and compared the proportion of PrP homozygous (individuals with two copies of same *PRNP* haplotypes) and heterozygous (individuals with two different *PRNP* haplotypes) using both sets of primers. Overall, by examining the composition and distribution, frequencies, and temporal changes in the genetic vulnerability of deer populations within these counties we sought to provide information for managers seeking to contain CWD within the state of Illinois.

## Methods

### Geographic locations of sampling counties

**Fig 1:**
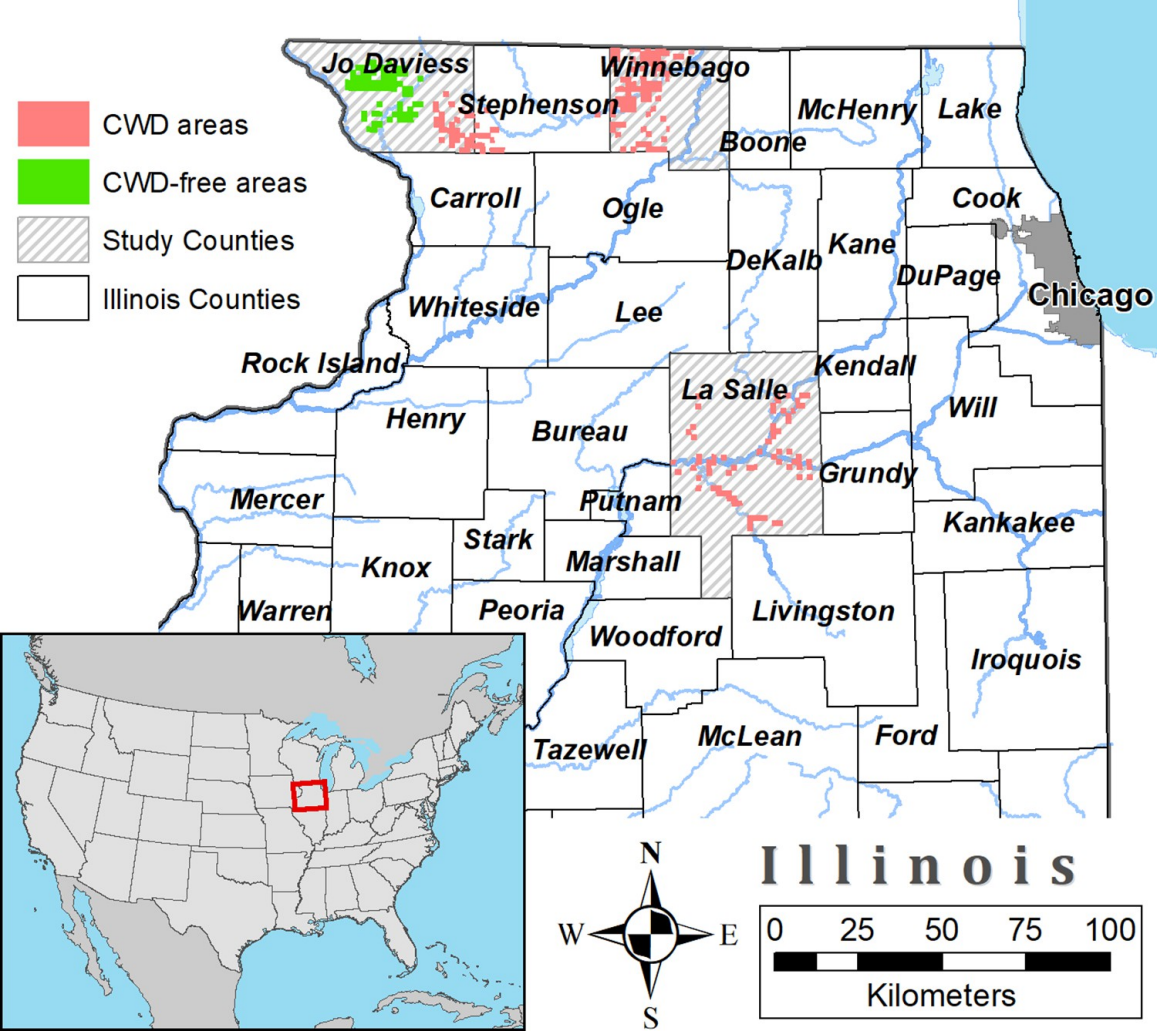
Location of the counties in Illinois and areas sampled in this study. Jo Daviess had its first case of CWD reported in Fiscal Year (FY) 2011. Winnebago has reported cases every FY since 2003. La Salle had a case in FY 2007 followed by several years of no reported CWD cases. After FY 2011 cases were reported every fiscal year. The Jo Daviess CWD cluster extended into nearby counties. This figure was generated using ESRI ArcMap 10.6 [31] using public domain base data.

Temporally, Illinois reports their cases of CWD in wild white-tailed deer by fiscal year (FY) which runs from July 1 of the year previous to the one designated, until June 30 of the designated year (thus FY 2007 represents the time frame from July 1 of 2006 until June 30 of 2007). Furthermore, we define wild white-tailed deer as a natural resource, not fenced-in white-tailed deer. Additionally, the state of Illinois has maintained a hunter-harvest CWD surveillance program since FY 2003 [28]. Jo Daviess, LaSalle, and Winnebago counties in Illinois were chosen for this study based on the historical reports of CWD and their geographic location (the counties are not contiguous to each other) (Fig 1, Table 1). Winnebago County is located at the northern Illinois border with Wisconsin, a state with CWD positive cases since FY 2002 [29]. The first confirmed CWD-positive deer in Winnebago County was in FY 2003. In FY 2003 there were a total of 3 CWD cases reported and thereafter there were 10 to 20 CWD reported cases per year up to FY 2011, with a declining number of reported CWD cases starting around FY 2012 [30] (Table 1). LaSalle County is in the north-central region of Illinois, but south of Jo Daviess and Winnebago. The county’s first confirmed CWD case was in FY 2007, with an undetected/low number of reported positive cases from FY 2008 to FY 2015, followed by an increase in reported CWD cases in FY 2019 and FY 2020 [35] (Table 1). At the border of Jo Daviess/Stephenson counties (south-east corner of Jo Daviess) there was a large increase in the CWD-positive deer reported in FY 2020, whereas for the same region from FY 2007 to FY 2012 there was one reported CWD positive deer. In the north-western corner of Jo Daviess County around the Galena Territory, there continue to be no reported CWD cases. This area (the north-western corner of Jo Daviess County) was compared to the Jo Daviess/Stephenson counties region reporting CWD cases in FY 2020. We used the same time frames for the comparisons between the two Jo Daviess regions.

**Table 1:** The number of confirmed CWD cases per hunter harvest samples tested from all sources for fiscal years 2003 to 2020 in the Illinois counties examined.

County	2003	2004	2005	2006	2007	2008	2009	2010	2011	2012	2013	2014	2015	2016	2017	2018	2019	2020
Jo Daviess	0/ 500	0/ 521	0/ 507	0/27	**0/56**	**0/ 112**	**0/62**	**0/ 201**	**1/ 228**	**0/ 1206**	1/ 1181	4/ 1066	7/ 1365	9/ 1387	10/ 1407	8/ 1471	12/ 1331	**25/ 1234**
Winnebago	3/ 394	20/ 984	13/ 1009	24/ 1091	17/ 1026	18/ 997	12/ 640	16/ 512	**10/ 409**	7/ 378	5/ 331	13/ 311	8/ 243	1/ 238	6/ 263	4/ 247	**9/ 289**	7/ 227
LaSalle	0/48	0/1	0/0	0/1	1/8	0/ 384	0/ 932	0/ 660	3/ 840	**0/ 663**	1/ 591	2/ 381	6/ 402	5/ 456	4/ 473	5/ 458	**6/ 544**	20/ 550

Bold indicates years during which deer for this study were sampled. Fiscal year = July 1 through June 30 of the following calendar year; In Jo Daviess, two regions (CWD free and CWD present) were sampled during the same periods. Data is represented as the number of confirmed CWD cases/number of samples tested.

### Sample selection

The Illinois Department of Natural Resources (IDNR) oversees the Illinois CWD surveillance and management program. Samples selected for this study were known CWD negative and obtained from deer that entered the hunter harvest surveillance efforts for CWD and from samples donated by hunters to our laboratory. CWD negative deer were selected because the proteotype AA is overrepresented in CWD positive deer and we wanted to choose samples in an unbiased manner. All samples processing within this work was approved by the University of Illinois Biosafety committee under IBC-33.2.

A total of 592 negative CWD hunter-harvested deer tissue samples (100 deer for each county/time frame for LaSalle and Winnebago counties, and 48 deer for Jo Daviess in the areas with CWD/CWD-free per time frame) were selected from the archived research samples at the Illinois Natural History Survey, University of Illinois. Sample selection consisted of negative CWD hunter-harvest deer that were randomly selected without prior knowledge of sex or age at the spatial scale of 1.6 x 1.6 km, township, range, and section (TRS) as defined by the Public Lands Survey System (United States Geological Survey (USGS)) and as previously defined within past Illinois *PRNP* studies [28, 32]. The TRS information was collected at the time of field sample collection during the surveillance program [28, 33]. A paired spatial sampling approach was used for all the counties. One hunter harvest sample was randomly chosen for as many TRS location as possible within each county at one time frame. The second sample from the second time frame was selected randomly from the same TRS as the previous timeframe. When no exact paired TRS sample could be obtained, a sample from an adjacent TRS was used to keep paired samples as spatially close as possible. All samples were derived from muscle tissue stored in 95% ethanol with yearly ethanol replacement.

### DNA extraction, amplification, and sequencing

DNA was extracted using the Qiagen DNeasy Blood & Tissue Kit (Qiagen Inc., Valencia CA). The complete *PRNP* coding region was amplified using the O’Rourke primers [14]: forward primer *PRNP*-223 (5’-ACACCCTCTTTATTTTGCAG-3’) and reverse primer *PRNP*-224 (5’-AGAAGATAATGAAAACAGGAAG-3’). These primers amplify the *PRNP* gene but not a pseudogene [14]. Total PCR volume was 25 μL (12.5 μL of GoTaq Green Master Mix (Promega, USA, WI), 1 μL of each 20 μM primer *PRNP*-223 and *PRNP*-224, 2 μL of DNA, and 8.5 μL DNA-free water). The following thermal cycle parameters were used: initial denaturation at 94°C for 5 minutes, followed by 36 cycles of 94°C for 25 seconds, 56°C for 30 seconds, 72°C for 1 minute with a final extension step of 72°C for 5 minutes. Gel electrophoresis (1% TBE agarose gel stained with ethidium bromide) was used to verify the presence of PCR product before purification with exonuclease I (New England Biolabs, USA, MA; 500 units at 1,000 units/mL) and shrimp alkaline phosphatase (New England Biolabs, USA, MA; 20,000 units/μL at 15000 units).

A BigDye® Terminator 3.1 cycle sequencing kit (Applied Biosystems Inc.) was used for the sequencing reaction using 0.6 μL of 2 μM primer for each direction. Sequences were separately generated for both forward and reverse PCR primers (*PRNP*-223, *PRNP*-224). In addition, previously published internal primers *PRNP*-IF (5’-ATGCTGGGAAGTGCCATGA-3’) and *PRNP*-IR (5’-CATGGCACTTCCCAGCAT-3’) were also used for sequencing to generate high-quality base calls across the entire length of the amplified fragment [18]. Sanger sequencing was performed on an Applied Biosystems 3730XL high-throughput capillary sequencer at the Roy J. Carver Biotechnology Center at the University of Illinois Urbana-Champaign. The resulting sequences were visually examined and edited after contig assembly using Sequencher 5.1 (Gene Codes Corporation, [34]) using the following settings: assembly algorithm (dirty), optimized gap placement (realigner and 3’ gap placement), minimum match percentage (85), and minimum overlap (20). Any individual sample with base call ambiguity was re-sequenced.

Because [27] recently reported that allelic dropouts may occur using O’Rourke primers [14], we repeated the PCR and sequencing steps to verify all homozygous genotypes using the Haley primers [27]: forward primer *WTDPRNP*-F (5’-TGTTTATAGCTGATGCCACTGC-3’) and reverse primer *WTDPRNP*-R (5’-ACACCACCACTACAGGGC-3’). All analyses were based on results in which the *PRNP* homozygotes were confirmed using the Haley primers. When a novel proteoform was inferred from the haplotype sequences (see below), the above procedures were repeated two additional times, with newly extracted DNA each time from the original tissue sample.

### Haplotype, diplotype, proteoform, and PrP proteotype inference and frequency calculation

The open reading frame was identified using NCBI Open Reading Frame Finder (https://www.ncbi.nlm.nih.gov/orffinder/), all sequences were aligned and trimmed to the open reading frame using MEGA6 [35] and exported as a FASTA file. Each FASTA file contained 100 deer for each county/time frame for LaSalle and Winnebago counties and 48 deer for Jo Daviess CWD/CWD-free area per time frame. Inferred haplotypes were based on each aforementioned FASTA file containing unphased sequences using PHASE with 10000 iterations and 100 burn-in iterations [18]. Both inferred haplotypes and protein translation was completed using DNA Sequence Polymorphism v6.12.03 (DnaSP6, [36]). Prion protein haplotype and PrP proteoform designations followed those of [17] and [18] respectively. Haplotype and PrP proteoform frequencies were calculated by taking the total number of haplotype or proteoform in each location/time and dividing it by the total number in the sample. Diplotype and PrP proteotype frequencies were calculated by taking the number of individuals with a particular diplotype or proteotype and dividing it by the total number of deer sampled per location/time.

### Statistical analyses using all haplotype and all diplotype data

For the haplotype and diplotype datasets, non-metric multidimensional scaling ordination using Bray–Curtis dissimilarity data matrix from frequency data [37] was completed to visualize the importance of location, and time (past vs. recent), using the ‘vegan’ package [38] in R statistical software version 4.1.2 [39] in RStudio v2021.09.1 [40]. Statistical significance was assessed with 1) ‘*envfit* function’ and 2) ‘*adonis’* using the ‘vegan’ package in R with 1000 permutations and 10 iterations with p-values reported as a range. The functions ‘*betadisper’* and ‘*permutest’* were used to test for multivariate homogeneity of groups dispersions. For ‘*betadisper’*, type was set to median, and *bias*.*adjust* set to true. For *permutest*, the number of permutations was set to 999 and the permutated p-values were reported.

### Statistical analyses using major haplotypes and PrP proteoforms

Due to the statistical limitations of the Chi-square test only major haplotypes and proteoforms were used. At the gene level, the major haplotypes were defined as haplotypes with a minimum frequency of 0.05 in any sampling county/time frame and consisted of haplotypes A through F. Haplotype E was removed due to the zero count for some analyses in Jo Davies CWD area FY 2007 –FY 2012. At the protein level, proteoforms A, C, and F were defined as major and analyzed because the other proteoforms’ were very low in frequency and their impact on CWD vulnerability is currently unknown. Haplotypes, proteoforms, and proteotypes frequency differences between/among counties and time frames were tested with Fisher’s exact test and/or Pearson’s Chi-squared test using R version 3.6.3 in RStudio version 1.3.1093. When there were no significant differences between counties, the counties were treated as strata and the Cochran-Mantel-Haenszel test was conducted in R statistical package. Prior to conducting the Cochran-Mantel-Haenszel test, we confirmed that the odds ratio across strata was not significantly different under the Woolf test using the ‘vcd’ package (v1.4–7; [41]). Benjamini-Hochberg procedure was completed for multiple comparisons in R statistical package.

### Prediction of non-synonymous amino acid substitutions

PrP proteoform L230 (A230T) was evaluated using PROVEAN (*Pro*tein *V*ariation *E*ffect *An*alyzer) web server (PROVEAN v1.1.3., provean.jciv.org, accessed January 31, 2022) to determine the effect of the non-synonymous amino acid substitution on protein function [42]. Prion protein proteoforms C, F, K, L, and U were also evaluated and compared to the results of [26]. A PROVEAN score of less than -2.5 indicates that the amino acid substitution is considered deleterious and a score above -2.5 is considered neutral.

## Results

### PRNP haplotypes

**Fig 2:**
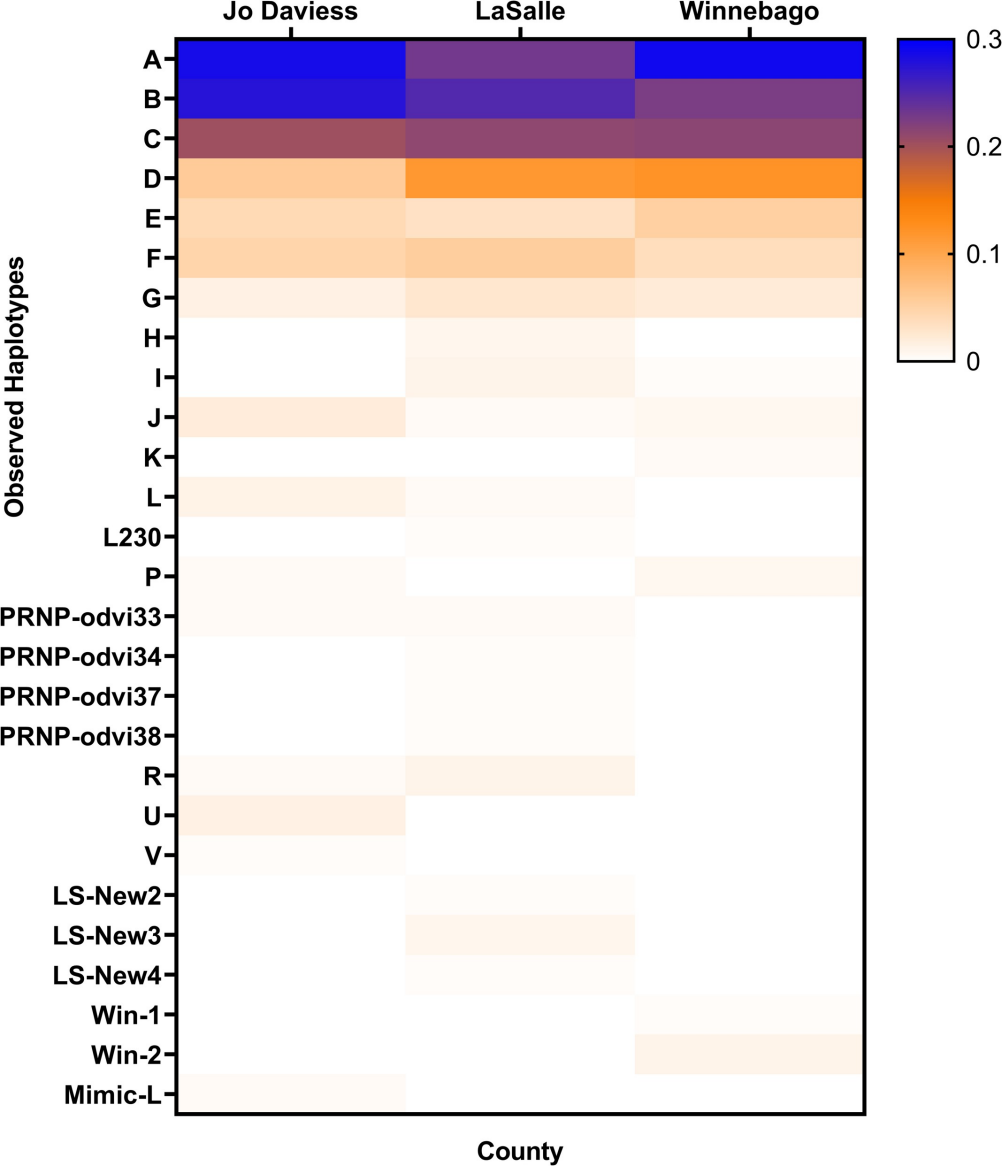
Heat map of *PRNP* haplotype frequencies found in Jo Daviess, LaSalle, and Winnebago counties from hunter-harvested CWD-negative deer. The color range indicates haplotype frequency based on the Haley et al. [27] primer combination. Data represents the average *PRNP* haplotype frequency of the two time frames.

Haplotypes data provides information about the different alleles within a population. In total, we sequenced 592 white-tailed deer individuals (S1 File). Twenty-seven *PRNP* haplotypes were inferred from the 592 coding sequences (Fig 2, S1 Table) across all white-tailed deer. White-tailed deer populations in Jo Daviess County carried on average 11 *PRNP* haplotypes (range 10–12) across different time frames and locations; in LaSalle County, 15 *PRNP* haplotypes on average (range 13–17); and in Winnebago County, 10.5 *PRNP* haplotypes on average (range 10–11) (S1 Table). In all counties regardless of year, the most common haplotype was A, followed by B and C (S1 Table). The six most common *PRNP* haplotypes (Haplotypes A, B, C, D, E, and F) accounted for 91.6% of all deer sampled. Haplotype L230 was found in one deer in LaSalle County, with L230 being the first report of this haplotype in the USA. Novel rare inferred haplotypes were found in Jo Daviess, LaSalle, and Winnebago counties, consisting of new combinations of previously identified single nucleotide polymorphisms.

Overall haplotype frequency differences among the three counties were partially supported by *envit* (10 iterations, *p* range = 0.041–0.061, *r*^2^ = 0.53) but not adonis (Fig 2, S1 Table). When comparing only the six major haplotypes (A through F), no significant spatial or temporal changes were detected (S2 Table). When comparing individual haplotype changes, there was a significant increase in haplotype F when compared to all other haplotypes in Winnebago County from 0.015 in 2011 to 0.06 in 2019 (S1 Table; Fisher’s exact test, two-tailed, *p* = 0.029).

### PRNP diplotype results

**Fig 3:**
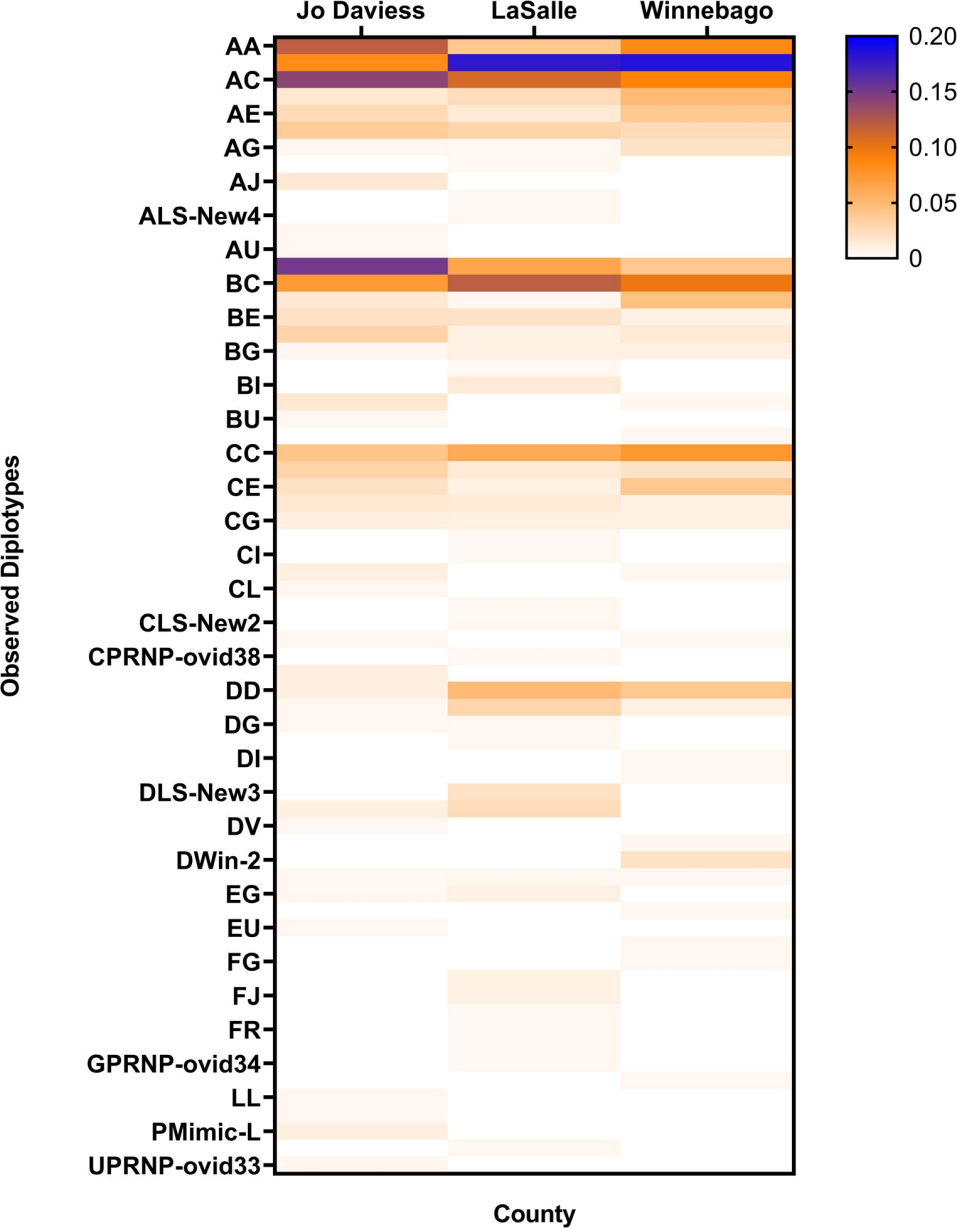
Heat map of the *PRNP* diplotype frequencies found in Jo Daviess, LaSalle, and Winnebago counties from hunter-harvested CWD-negative deer. The color range indicates diplotype frequency based on the Haley primer combination. Data represents the average *PRNP* haplotype frequency of the two time frames.

Diplotype data provides information about the haplotype make-up of each individual and the frequency of which individuals carry the same two haplotypes. Sixty-seven diplotypes were identified with the six most frequent across all deer identified as diplotypes AB (0.150), AC (0.113), BC (0.098), BB (0.084), AA (0.081), and CC (0.059) (Fig 3, S3 Table.) Jo Daviess white-tailed deer populations had an average of 20 (range: 18–23) diplotypes, LaSalle on average 29 (range: 25–33), and Winnebago on average 20 (range: 18–23) diplotypes. Each county had several unique diplotypes (Fig 3, S3 Table), and county-wide diplotype frequencies had significant beta dispersion (betadisper; permutest, *df* = 2, *F* = 6.67, *p* = 0.001). Pairwise comparison indicated that LaSalle and Jo Daviess counties had similar diplotype beta dispersion to each other as compared to Winnebago county (LaSalle vs. Winnebago, *p* = 0.007 and Jo Daviess vs. Winnebago, *p* = 0.011) indicating that the community structure (overall diplotype frequencies) in Winnebago is different from the other two counties.

### PrP proteoform results

Proteoform data provides information about the protein variation within a population. PrP proteoforms were determined for each *PRNP* haplotype. Seven PrP proteoforms in total were detected in Jo Daviess, LaSalle, and Winnebago counties. Proteoform A was the most frequent (70.6% of total) followed by proteoform C (23.3% of total, Table 2) per sample location/time point. Prion protein proteoform L230 was identified in the USA for the first time in white-tailed deer. Results from PROVEAN indicated that PrP proteoform L230 was neutral (Table 2), while all other PrP proteoforms PROVEAN predictions were identical to previously published research.

**Table 2:** Frequency of PrP proteoforms found in white-tailed deer in Jo Daviess, LaSalle, and Winnebago counties, Illinois.

Protein proteoform^a^	AA	LS FY 2012	LS FY 2019	Win FY 2011	Win FY 2019	JD CWD free FY 2007 –FY 2012	JD CWD free FY 2020	JD CWD area FY 2007 –FY 2012	JD CWD area FY 2020	Total	PROVEAN prediction
A	^C^	0.705	0.685	0.755	0.690	0.740	0.740	0.729	0.594	0.706	not applicable
C	G96S	0.26	0.23	0.220	0.250	0.208	0.177	0.188	0.302	0.233	-1.25 neutral
F	Q95H	0.03	0.075	0.015	0.060*	0.010	0.042	0.052	0.083	0.046	-1.75 neutral
K	Q226K	nd	nd	0.010	nd	nd	nd	nd	nd	0.002	-0.51 neutral
L	A123T	0.005	0.005	nd	nd	0.010	0.021	0.021	0.021	0.008	-1.42 neutral
L230^b^	Q230L	nd	0.005	nd	nd	nd	nd	nd	nd	0.001	-1.43 neutral
U	N103I	nd	nd	nd	nd	0.031	0.021	0.010	nd	0.005	-2.73 deleterious

*Winnebago FY 2010 to Winnebago FY 2019 showed a significant increase in the frequency of PrP proteoform F

^a^ PrP proteoform designations are consistent with those of Ishida et al. 2020

^b^ PrP proteoform previously identified only from Canada; nd = not detected

^c^ Proteoform A amino acid sequence is considered wild type: Q95, G96, S100, N103, A123, Q226, Q230; LS = LaSalle; Win = Winnebago; JD = Jo Daviess. Total is the frequency of each prion protein proteoform in the entire sample pool. PROVEAN prediction indicates deleterious mutations will affect protein function. Deleterious mutations tend to be eliminated through selection while neutral mutations will remain within a population. Frequencies are based on Haley primer results.

The evaluation of the major proteoforms (A, C, and F) indicated that there was a significant difference in the PrP proteoform frequencies between Winnebago FY 2011 vs. Winnebago FY 2019 (3x2 contingency table, Fisher’s exact test: *p* = 0.039 after Benjamini-Hochberg adjustment, Pearson’s Chi-squared test: *X*^*2*^ = 6.3579, *df* = 2, *p* = 0.04). Compared to the PrP proteoform A, the frequency of PrP proteoform F significantly increased from 0.015 (*n* = 3) in FY 2011 to 0.06 (*n* = 12) in FY 2019 (Fisher’s exact test: *p* = 0.034 after Benjamini-Hochberg adjustment, OR = 4.36, 95% CI: 1.143–24.56) while the frequency of proteoform C was not significantly different (Fisher’s exact test: *p* = 0.41 after Benjamini-Hochberg adjustment, OR = 4.36, 95% CI: 0.756–2.04). Additionally, there was a significant difference in the major PrP proteoform frequencies (A, C, F) when we compared Jo Daviess CWD free area FY 2020 and the Jo Daviess CWD area FY 2020 (3x2 contingency table, Fisher’s exact test: p = 0.049 after Benjamini-Hochberg adjustment, Pearson’s Chi-squared test: *X*^*2*^ = 5.9742, *df* = 2, *p* = 0.05), and Winnebago FY 2011 was significantly significant as compared to Jo Daviess FY 2020, Pearson’s Chi-squared test: *X*^*2*^ = 6.461, *df* = 2, *p* = 0.04). All additional county and temporal comparisons based on the major proteoforms (A, C, F) were not significant (see S2 Table).

### White-tailed deer vulnerability results

**Fig 4:**
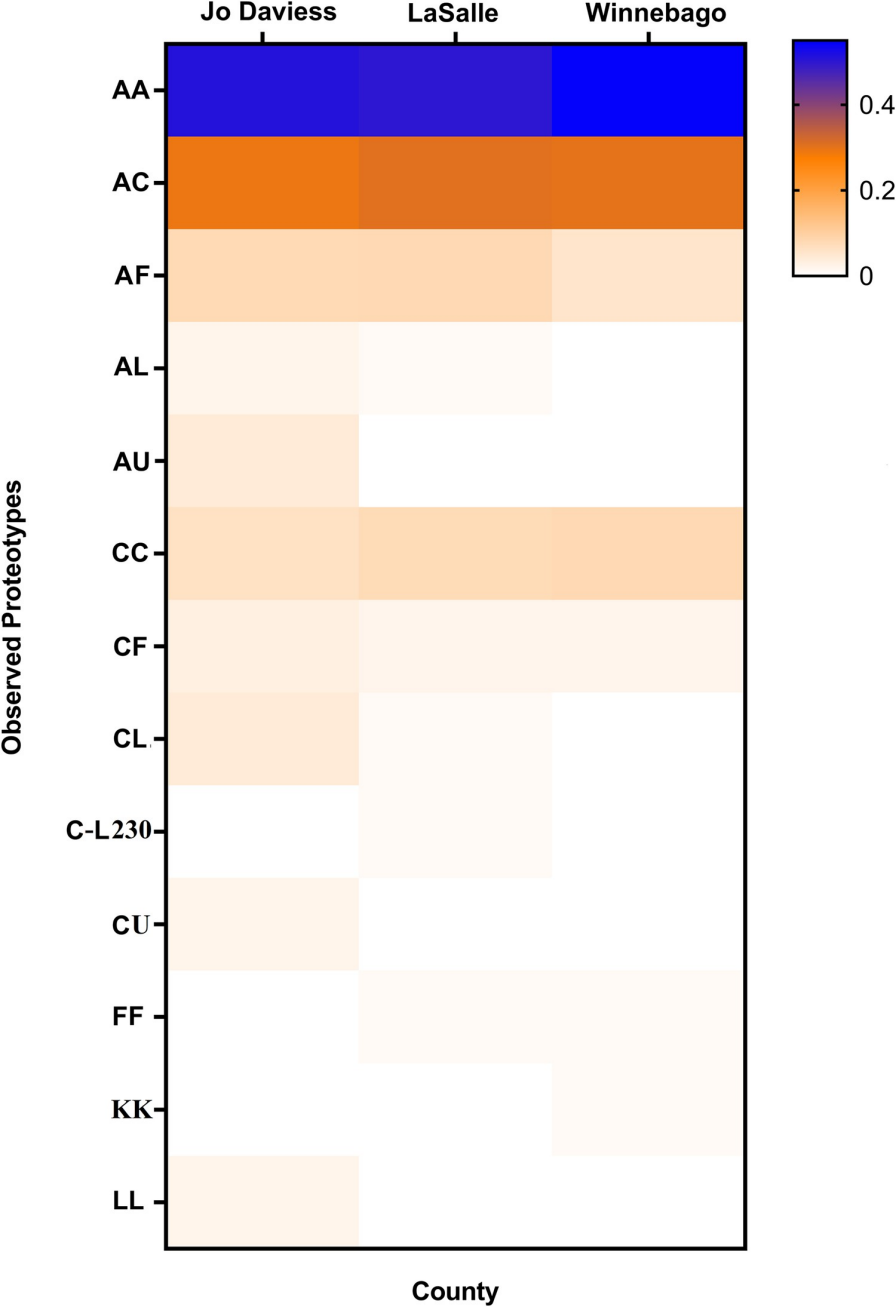
Heat map of PrP proteotype frequencies among white-tailed deer in Jo Daviess, LaSalle, and Winnebago counties, Illinois. The color range indicates proteotype frequency based on the Haley primer combination. Data represents the average *PRNP* haplotype frequency of the two time frames.

Proteotype data provides important information about how the proteoforms are spread across individuals within a population. In total, Illinois white-tailed deer showed 13 different PrP proteotypes (Fig 4, S4 Table). Most white-tailed deer carried haplotypes in both chromosomes that each encoded the most common PrP proteoform A (proteotype AA total frequency was 0.519). The second most common were the deer carrying haplotypes that encoded for the PrP proteoforms A and C (proteotype AC frequency was 0.299) (Table 3). Given that deer carrying at least one chromosome encoding proteoform C or F are less common among CWD-positive deer than among CWD-negative deer, we designated vulnerable deer as those in which both chromosomes encode PrP proteoform A (proteotype AA); while designating as less vulnerable deer if they carry at least one copy of proteoform C or F (i.e., PrP proteotypes AC, AF, CC, CF, and FF) (Table 4).

**Table 3:** White-tailed deer vulnerability in Jo Daviess, LaSalle, and Winnebago counties, Illinois.

White-tailed deer vulnerability	LS FY12	LS FY19	Win FY11	Win FY19	JD CWD free FY07 –FY12	JD CWD free FY20	JD CWD area FY07 –FY12	JD CWD area FY20	Total
Vulnerable	0.510 (51)	0.490 (49)	0.610 (61)	0.490 (49)	0.604 (29)	0.563 (27)	0.563 (27)	0.333 (16)	0.522
Less vulnerable	0.480 (48)	0.500 (50)	0.390 (39)	0.510 (51)	0.333 (16)	0.417 (20)	0.437 (21)	0.646 (31)	0.466
Unknown	0.010 (1)	0.010 (1)	nd	nd	0.063 (3)	0.021 (1)	nd	0.021 (1)	0.012

Vulnerable deer are those in which both chromosomes encode PrP proteoform A; while less vulnerable deer encode at least one copy of proteoform C or F; nd = not detected; JD = Jo Daviess; LS = LaSalle; Win = Winnebago. Data are shown as frequency (number of individuals). Total is the frequency of deer per category in the entire sample pool. Frequencies are based on Haley primer results.

**Table 4:** The number of homozygous white-tailed deer and the resulting diplotype change based on primer combinations.

Diplotype based on O’Rourke primers	# of homozygous deer with O’Rourke primers	# of homozygous deer with Haley primers	Diplotype change based on Haley primers
AA	57	47	AC, AF, A&new-4
BB	60	50	BC, BI, B&PRNP-Odvi37, BV
CC	40	35	BC, CD
DD	23	20	CD, DF, DI
EE	6	3	CE, EP,
FF	5	1	CF, IF
GG	2	1	G&PRNP-odvi34
KK	1	1	no change
LL	2	1	LP
LNew1-Mimic L	1	1	no change
PRNP-odvi33PRNP-odvi33	1	1	no change
Total	198	161	

Within the CWD area in Jo Daviess, there was a significant temporal decrease in the proportion of vulnerable deer in the Jo Daviess CWD area FY 2020 as compared to the same area in FY 2007– FY 2012 (Fisher’s exact test: *p* = 0.02 after Benjamini-Hochberg adjustment, OR = 2.87, 95% CI: 1.15–7.42) (Table 4). There was also a geographic difference, as the Jo Daviess CWD area in FY 2020 had more deer carrying protective PrP proteotypes as compared to the Jo Daviess CWD free area (Fisher’s exact test: *p* = 0.01 after Benjamini-Hochberg adjustment, OR = 0.33, 95% CI: 0.13–0.83) (Table 3). No additional significant differences were detected between counties and time frames (S2 Table).

### Primer comparison

The O’Rourke primers used to amplify *PRNP* in previous studies have recently been found to show allelic dropout in some cases. Thus, for this study, all homozygous deer in our sampling were verified using recently published Haley primers designed to avoid this allelic dropout. In total, 33% of deer analyzed were putatively homozygous (198 individuals) using the O’Rourke primers and 27% of individuals were homozygous (161 individuals) using the Haley primers. Changes in inferred frequencies between O’Rourke and Haley primer pairs are found in Fig 4, S5 Table, while S5 Table indicated the specific changes based on inferred *PRNP* diplotype.

**Fig 5:**
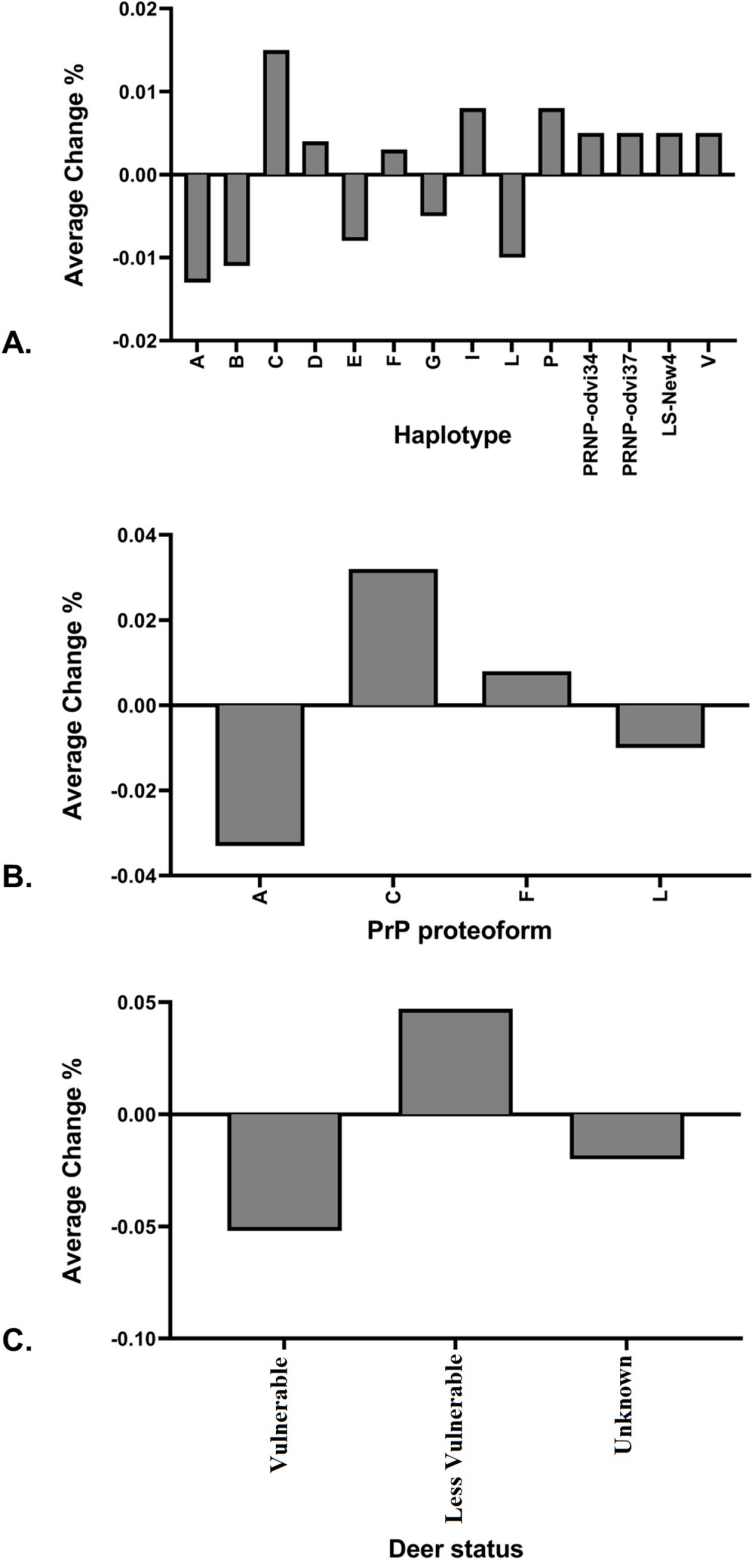
The change in observed frequencies of *PRNP*, PrP proteoform, and deer susceptibility between O’Rourke primers and Haley primers. (A) *PRNP* haplotype, (B) PrP proteoform, and (C) deer susceptibility. Frequencies are calculated by taking the observed frequency based on Haley primers minus the observed frequency based on O’Rourke primers.

Haplotype A had an average frequency reduction of 0.013, haplotype B had an average frequency reduction of 0.011, and haplotype C had an average frequency increase of 0.015 across all sites/timeframe (Fig 5A). Concerning the PrP proteoforms, proteoform A had an average frequency decrease by 0.033, and proteoform C had an average frequency increase by 0.032 (Fig 5B). The frequency of white-tailed deer that are deemed most susceptible (PrP proteotype AA) decreased on average by 0.052 and individual deer with at least one copy of an allele that codes for a more protective proteoform increased in frequency by 0.047 (Fig 5C).

## Discussion

Few studies have investigated *PRNP* and PrP frequencies in wild white-tailed deer at the county level. Research showed that white-tailed deer populations within the Mid-Atlantic states have *PRNP* variation at a fine geographic scale (sub-region and county level) [25], while [26] did not find evidence for geographical structure in the *PRNP* of Nebraska white-tailed and mule deer (*O*. *virginianus and O*. *hemionus* respectively). Our results appear to be consistent with those of [26] in that there was not much evidence for geographical structure in the *PRNP* of white-tailed deer among the three counties examined. We did find that Winnebago county had different diplotype beta-dispersion overall as compared to LaSalle and Jo Daviess counties suggesting some differences in diplotype frequencies (Fig 3, S3 Table), and we did detect novel rare haplotypes (Fig 2, S1 Table) not previously recorded. In addition, our data suggest that some rare haplotypes may have some localized distribution since they were only detected in one county. For example, rare Haplotype U was only found in Jo Daviess (detected in 3 of the 4 location/time sampling points), and rare Haplotype L was consistently found in LaSalle and Jo Daviess counties but not detected in Winnebago. Additionally, three new non-synonymous combinations for Illinois were identified only in LaSalle County and two new non-synonymous combinations for Illinois were identified only in Winnebago County. However, the findings are new and additional sampling in these counties and across the state of Illinois would be needed to clarify their distributional occurrence and relevance in relation to CWD.

In addition, we evaluated a subset of the haplotypes (A, C, and F) because they have been associated with CWD infection [17, 18, 28]. Based on the subset of data, our temporal comparisons indicated a general increase in the frequency in all three counties for the protective haplotype F, although the only county with a statistically significant increase was Winnebago. Although we do not know the exact reason for an increase in the haplotype F, one plausible explanation may be the regional migration of deer into Illinois from Wisconsin. Illinois’ Winnebago County borders Wisconsin, which has experienced a much greater prevalence of CWD, with some areas reporting having 43–56% CWD infection rates for male white-tailed deer and 23–35% infection rates for female deer [43]. The increase in less vulnerable deer in Illinois may therefore reflect gene flow between deer populations in Illinois and Wisconsin, with selection in Wisconsin potentially also affecting, through migration and gene flow, the Illinois deer population. Further studies would be required to support this hypothesis.

This study also investigated the CWD vulnerability of Illinois white-tailed deer within the three counties by examining across time the proportion of deer with proteotypes that are more or less vulnerable to CWD. All counties showed a decline in the proportion of vulnerable deer, although the only decline in frequency that was statistically significant was in the Jo Davies CWD area in FY 2020 (Table 3). This could potentially be impacted by CWD management which can remove vulnerable white-tailed deer from CWD infected areas [32].

Several novel haplotypes were found in this study in all three counties, of which only haplotype L230 had a non-synonymous mutation in the *PRNP* not previously reported within Illinois. Haplotype L230 was previously reported from Alberta and Saskatchewan, Canada [44] but this is the first report of the *PRNP* haplotype within the USA, likely due to being a rare haplotype requiring a large number of samplings prior to detection. Using PROVEAN, the PrP proteoform L230 is predicted to be neutral to the function of the protein and the other PrP proteoforms found in this study had identical predictions as [26]. Continued investigation across central and southern counties may find additional novel *PRNP* haplotypes. While PrP proteoform L230 is predicted to be neutral to the function of the protein, reporting the occurrence of novel haplotypes is valuable as we continue to develop our understanding of their role in the occurrence of CWD. Nonetheless, the importance of rare haplotypes is unclear, and only increased evaluation of these haplotypes in known CWD infected, or negative deer remains to be evaluated.

In addition, we demonstrate that correctly identifying heterozygous and homozygous individuals is critical for the precise understanding of the level of vulnerability to CWD in a population. Among deer identified as *PRNP* homozygous using the O’Rourke primers, 18.7% were found to be heterozygous when amplified and sequenced with the newly designed Haley primers. Only 8% of deer were affected by the allelic dropout using the O’Rourke primers. This is a small proportion that would have produced a slight inaccuracy but would not call into question the overall conclusions of previous *PRNP* studies in the state of Illinois. However, the new primer pair would be recommended for future studies of *PRNP* and PrP proteotype profiles because it appears to provide more accurate frequency estimates. Further evaluation of the Haley primer pair will provide a more robust understanding of how well these primers amplify the different *PRNP* genotypes.

A major strength of this study design was that by using the hunter-harvest CWD negative samples, we were able to sample a larger geographical area in a county compared to just using samples from the localized culling program in Illinois where the samples come from a localized and focal area where CWD has been detected [45]. We believe this approach allowed for a more accurate representation of the county-level white-tailed *PRNP* frequencies. Lastly, the study design allowed us to investigate frequencies at the gene, protein, and individual deer level using two published primer sets to account for potential allelic dropout [14, 27]. Understanding the frequency of protective alleles in a population is an important aspect of understanding the potential for a CWD outbreak, but it is also important to understand the distribution of those alleles across individuals.

In summary, Illinois counties were examined for spatial and temporal differences in *PRNP* haplotype frequencies, PrP proteoform frequencies, and the frequencies of susceptible deer. Furthermore, we looked at the trends and differences in the main haplotypes A through F because they have been associated with CWD-positive or CWD-negative deer. It is important to note that all counties retained a large frequency (range: 0.333–0.563) of the PrP proteotype AA deer that are highly vulnerable to CWD in the most recent timeframes (FY2019-2020, S4 Table).

We also demonstrated that the primer pair developed by [27] identified that 18.7% of the homozygous individuals, as identified by the [20] primer pair, were heterozygous (S5 Table). Importantly, because the changes in calculated *PRNP* haplotype and PrP proteoform frequencies were minor (on average: PRNP < 0.02, PrP proteoform < 0.04, and PrP proteotype < 0.06, Fig 5A–5C, S5 Table) following the use of the [27] primer pair, the conclusions of previous studies that compared CWD status with *PRNP* or PrP are not called into question. Overall, understanding the spatial distribution of variants of *PRNP* and PrP, and temporal changes in those frequencies will be essential to guide management and to build on efforts to contain the spread of CWD within the state of Illinois.

## Supporting information

S1 File Final resulting *PRNP* haplotype sequence assigned to each individual deer. (TXT)

S1 Table The frequency of the PRNP haplotypes among hunter-harvested CWD-negative white-tailed deer in Jo Daviess, LaSalle, and Winnebago counties, Illinois. Only polymorphic sites are shown; all dots indicate the same nucleotide as haplotype A; nd = not detected; grey shading indicates non-synonymous nucleotide substitutions; past = 2007–2012; JD = Jo Daviess; LS = LaSalle; Win = Winnebago; Total is the frequency of each haplotype in the entire sample pool; Frequencies are based on Haley primers.
(DOCX)

S2 Table *PRNP* Haplotype, *PrP* proteoform, and *PrP* proteotype analyses; marginal or non-statistical results. ^1^Results from the *envif* and adonis are unreliable due to significant beta dispersion.
(DOCX)

S3 Table The frequency of the diplotypes found in hunter-harvest white-tailed deer samples from Jo Daviess, LaSalle, and Winnebago Counties, Illinois. nd = not detected; past = 2007–2012; JD = Jo Daviess; LS = LaSalle; Win = Winnebago; total unique diplotypes = 67; Diplotypes based on Haley et al. 2021 primers; deer with the haplotypes AA are highly vulnerable to CWD; that deer with a CC or an FF are considered to have a lower vulnerability to CWD; that deer with an AC, AF are less vulnerable than AA alone; Total is the frequency of each diplotype in the entire sample pool; Frequencies are based on Haley primers.
(DOCX)

S4 Table PrP proteotype frequencies among white-tailed deer in Jo Daviess, LaSalle, and Winnebago counties, Illinois. (DOCX)

S5 Table The change in observed frequencies of *PRNP*, PrP proteoform, and deer susceptibility between O’Rourke primers and Haley primers. (A) *PRNP* haplotype, (B) PrP proteoform, and (C) deer susceptibility. Frequencies are calculated by taking the observed frequency based on Haley primers minus the observed frequency based on O’Rourke primers.
(DOCX)
