# Supplementary material for: County-wide assessments of Illinois white-tailed deer (Odocoileus virginianus) prion protein gene variation using improved primers and potential implications for management
Source: PLoS One. 2022 Nov 30;17(11):e0274640. doi: 10.1371/journal.pone.0274640 (PMC9710747; doi:10.1371/journal.pone.0274640)
Supplement: S1 Table — Only polymorphic sites are shown; all dots indicate the same nucleotide as haplotype A; nd = not detected; grey shading indicates non-synonymous nucleotide substitutions; past = 2007–2012; JD = Jo Daviess; LS = LaSalle; Win = Winnebago; Total is the frequency of each haplotype in the entire sample pool; Frequencies are based on Haley primers. (DOCX) [file pone.0274640.s002.docx]

| Haplotype | Nucleotide location | | | | | | | | | | | | | | | Sampling location and year | | | | | | | | |  | |
| --- | --- | --- | --- | --- | --- | --- | --- | --- | --- | --- | --- | --- | --- | --- | --- | --- | --- | --- | --- | --- | --- | --- | --- | --- | --- | --- |
|  | 060 | | 153 | 243 | 285 | 286 | 308 | 324 | 367 | 372 | 438 | 555 | 676 | 689 | LS FY12 | | LS  FY19 | Win  FY11 | Win  FY19 | JD CWD free FY07 – FY12 | JD CWD free FY20 | JD CWD area FY07 – FY12 | JD CWD area FY20 | Total | |  |
| A | C | C | | T | A | G | A | A | G | G | C | C | C | A | 0.255 | | 0.205 | 0.305 | 0.275 | 0.260 | 0.281 | 0.313 | 0.292 | 0.268 | |  |
| B | . | . | | . | . | . | . | . | . | . | . | T | . | . | 0.270 | | 0.230 | 0.240 | 0.210 | 0.344 | 0.240 | 0.313 | 0.208 | 0.250 | |  |
| C | . | . | | . | . | A | . | . | . | . | . | T | . | . | 0.235 | | 0.190 | 0.215 | 0.215 | 0.208 | 0.177 | 0.177 | 0.250 | 0.210 | |  |
| D | . | T | | . | . | . | . | . | . | . | . | . | . | . | 0.100 | | 0.135 | 0.110 | 0.135 | 0.063 | 0.063 | 0.063 | 0.042 | 0.100 | |  |
| E | . | . | | . | . | . | . | . | . | . | . | . | . | . | 0.015 | | 0.050 | 0.055 | 0.050 | 0.031 | 0.094 | nd | 0.042 | 0.042 | |  |
| F | T | . | | . | C | . | . | . | . | . | . | . | . | . | 0.040 | | 0.070 | 0.015 | 0.060 | 0.010 | 0.042 | 0.052 | 0.083 | 0.046 | |  |
| G | T | . | | . | . | . | . | . | . | . | . | . | . | . | 0.020 | | 0.035 | 0.030 | 0.015 | nd | 0.021 | 0.021 | 0.021 | 0.022 | |  |
| H | . | . | | . | . | . | . | . | . | A | . | T | . | . | nd | | 0.020 | nd | nd | nd | nd | nd | nd | 0.003 | |  |
| I | . | . | | A | . | A | . | . | . | . | . | T | . | . | 0.010 | | 0.015 | nd | 0.005 | nd | nd | nd | nd | 0.005 | |  |
| J | . | . | | . | . | . | . | G | . | . | . | . | . | . | 0.010 | | nd | 0.015 | nd | 0.010 | 0.031 | 0.021 | 0.021 | 0.011 | |  |
| K | T | . | | . | . | . | . | . | . | . | . | . | A | . | nd | | nd | 0.010 | nd | nd | nd | nd | nd | 0.002 | |  |
| L | . | . | | . | . | . | . | . | A | . | . | . | . | . | 0.005 | | 0.005 | nd | nd | nd | 0.010 | 0.021 | 0.021 | 0.006 | |  |
| L230 | T | . | | . | . | . | . | . | . | . | . | . | . | T | nd | | 0.005 | nd | nd | nd | nd | nd | nd | 0.001 | |  |
| P | . | . | | . | . | A | . | . | . | . | . | . | . | . | nd | | nd | 0.005 | 0.010 | nd | nd | 0.010 | 0.010 | 0.004 | |  |
| PRNP-odvi33 | . | T | | . | . | . | . | . | . | . | T | . | . | . | nd | | 0.010 | nd | nd | 0.021 | nd | nd | nd | 0.003 | |  |
| PRNP-odvi34 | T | . | | . | . | A | . | . | . | . | . | . | . | . | nd | | 0.005 | nd | nd | nd | nd | nd | nd | 0.001 | |  |
| PRNP-odvi37 | T | T | | . | C | . | . | . | . | . | . | . | . | . | 0.005 | | nd | nd | nd | nd | nd | nd | nd | 0.001 | |  |
| PRNP-odvi38 | T | . | | . | . | . | . | . | . | . | . | T | . | . | Nd | | 0.005 | nd | nd | nd | nd | nd | nd | 0.001 | |  |
| R | . | T | | . | . | . | . | . | . | . | . | T | . | . | 0.025 | | nd | nd | nd | 0.010 | 0.010 | nd | nd | 0.006 | |  |
| U | . | T | | . | . | . | T | . | . | . | . | . | . | . | nd | | nd | nd | nd | 0.031 | 0.021 | 0.010 | nd | 0.005 | |  |
| V | . | T | | . | . | A | . | . | . | . | . | . | . | . | nd | | nd | nd | nd | nd | nd | nd | 0.010 | 0.001 | |  |
| LS-New2 | . | . | | . | C | . | . | . | . | . | . | T | . | . | nd | | 0.005 | nd | nd | nd | nd | nd | nd | 0.001 | |  |
| LS-New3 | . | . | | . | . | A | . | . | . | . | . | T | . | . | 0.010 | | 0.010 | nd | nd | nd | nd | nd | nd | 0.003 | |  |
| LS-New4 | . | . | | . | . | A | . | G | . | . | . | . | . | . | nd | | 0.005 | nd | nd | nd | nd | nd | nd | 0.001 | |  |
| Win-1 | . | . | | . | . | . | T | . | . | . | . | T | . | . | nd | | nd | nd | 0.005 | nd | nd | nd | nd | 0.001 | |  |
| Win-2 | . | T | | . | . | A | . | . | . | . | . | T | . | . | nd | | nd | nd | 0.025 | nd | nd | nd | nd | 0.004 | |  |
| Mimic-L | . | T | | . | . | . | . | . | A | . | . | . | . | . | nd | | nd | nd | nd | 0.010 | 0.010 | nd | nd | 0.002 | |  |
