# Supplementary material for: County-wide assessments of Illinois white-tailed deer (Odocoileus virginianus) prion protein gene variation using improved primers and potential implications for management
Source: PLoS One. 2022 Nov 30;17(11):e0274640. doi: 10.1371/journal.pone.0274640 (PMC9710747; doi:10.1371/journal.pone.0274640)
Supplement: S2 Table — 1Results from the envif and adonis are unreliable due to significant beta dispersion. (DOCX) [file pone.0274640.s003.docx]

|  | Comparison | | Statistical test | *M^2^* | *X^2^* | *df* | *p* value | r^2^ |
| --- | --- | --- | --- | --- | --- | --- | --- | --- |
| **Full dataset analyses** | |  | |  |  |  |  |  |
| **Haplotypes** | |  | |  |  |  |  |  |
| County differences | | *envif* | | - | - | - | 0.04-0.06 | 0.53 |
|  | | *adonis* | | - | - | 2 | 0.09-0.11 | 0.40 |
|  | | betadisper: type=median, bias.adjust=true, ANOVA | | - | - | 2 | 0.24 | - |
| **Diplotypes^1^** | | *envif* | | - | - | - | 0.01-.03 | 0.51 |
| County differences | | *adonis* | | - | - | - | 0.03 | 0.54 |
|  | | betadisper: type=median, bias.adjust=true, ANOVA | | - | - | 2 | 0.001 | - |
| LaSalle FY12 vs. Winnebago FY11 | | 6 x 2 cont. table, Fisher’s | | - | - | - | 0.14 | - |
|  | | Pearson’s Chi-square | | - | 8.27 | 5 | 0.14 | - |
| LaSalle FY12 vs. Jo Daviess CWD area FY07-FY12 | | 5 x 2 cont. table, Fisher’s | | - | - | - | 0.49 | - |
|  |  | Pearson’s Chi-square | | - | 3.42 | 4 | 0.49 | - |
| LaSalle FY19 vs. Jo Daviess CWD FY20 | | 6 x 2 cont. table, Fisher’s | | - | - | - | 0.09 | - |
|  |  | Pearson’s Chi-square | | - | 9.03 | 5 | 0.11 | - |
| Jo Daviess CWD free FY07-FY12 vs. Jo Daviess CWD area FY07-FY12 | | 5 x 2 cont. table, Fisher’s | | - | - | - | 0.51 | - |
|  |  | Pearson’s Chi-square | | - | 3.46 | 4 | 0.48 | - |
| Jo Daviess CWD free FY19 vs. Jo Daviess CWD area FY20 | | 6 x 2 cont. table, Fisher’s | | - | - | - | 0.42 | - |
|  |  | Pearson’s Chi-square | | - | 5.06 | 5 | 0.41 | - |
| **Major haplotypes (A-F) - Temporal** | |  | |  |  |  |  |  |
| LaSalle and Jo Daviess FY07-FY12 vs. LaSalle and Jo Daviess FY19-FY20 (treated as strata) | | Cochran Mantel-Haenszel | | 3.45 | - | 4 | 0.49 | - |
| Winnebago FY11 vs. Winnebago FY19 | | 6 x 2 cont. table, Fisher’s | | - | - | - | 0.24 | - |
|  | | Pearson’s Chi-square | | - | 6.67 | 5 | 0.25 | - |
| **Major haplotypes (A-D, F) - County** | |  | |  |  |  |  |  |
| Winnebago FY19 vs. Jo Daviess FY20 | | Pearson’s Chi-square | | - | 6.94 | 4 | 0.14 | - |
| Winnebago FY11 vs. Jo Daviess FY07-FY12 | | Pearson’s Chi-square | | - | 6.68 | 4 | 0.15 | - |
| **Proteoform (A, C, F) - County**  LaSalle FY12 vs. Winnebago FY11 | | 3 x 2 cont. table, Fisher’s  Pearson’s Chi-square | |  | -  2.01 | -  2 | 0.39  0.37 | -  - |
|  |  |  |  | - |  |  |  |  |
| LaSalle FY12 vs. Jo Daviess CWD area FY07-FY12 | | 3 x2 cont. table, Fisher’s | | - | - | - | 0.34 | - |
|  | | Pearson’s Chi-square | | - | 2.32 | 2 | 0.31 | - |
| LaSalle FY19 vs. Jo Daviess CWD area FY20 | | 3 x2 cont. table, Fisher’s | | - | - | - | 0.33 | - |
|  | | Pearson’s Chi-square | | - | 2.21 | 2 | 0.33 | - |
| Jo Daviess CWD free FY07-FY12 vs. Jo Daviess CWD area FY07-FY12 | | 3 x 2 cont. table, Fisher’s | | - | - | - | 0.32 | - |
|  | | Pearson’s Chi-square | | - | 2.77 | 2 | 0.25 | - |
| Winnebago FY11 vs. Jo Daviess FY07-FY12 | | Pearson’s Chi-square | | - | 1.34 | 2 | 0.50 | - |
| Winnebago FY19 vs. Jo Daviess FY20 | | Pearson’s Chi-square | | - | 0.20 | 2 | 0.90 | - |
| **Proteoform (A vs. C)** | |  | |  |  |  |  |  |
| Jo Daviess CWD free FY19 vs. Jo Daviess CWD area FY20 | | Fisher’s | | - | - | - | 0. 04 | - |
|  | | p-value adjust: Benjamini-Hochberg | | - | - | - | 0.08 | - |
| **Proteoform (A vs. F)** | |  | |  |  |  |  |  |
| Jo Daviess CWD free FY19 vs. Jo Daviess CWD area FY20 | | Fisher’s | | - | - | - | 0.37 | - |
| **Proteoform (A, C, F) - Temporal** | |  | |  |  |  |  |  |
| LaSalle and Jo Daviess FY07-FY12 vs. LaSalle and Jo Daviess FY19-FY20 (treated as strata) | | Cochran Mantel-Haenszel | | 5.10 | - | 2 | 0.08 | - |
| Winnebago FY11 vs. Jo Daviess FY20 | | Pearson’s Chi-square | | - | 3.09 | 2 | 0.21 | - |
| **Proteoform A vs. F - Temporal** | |  | |  |  |  |  |  |
| LaSalle and Jo Daviess FY07-FY12 vs. LaSalle and Jo Daviess FY19-FY20 (treated as strata) | | Cochran Mantel-Haenszel | | 4.27 | - | 1 | 0.04 | - |
|  | | p-value adjust: Benjamini-Hochberg | | - | - | - | 0.08 | - |
| **Proteoform A vs. C - Temporal** | |  | |  |  |  |  |  |
| LaSalle and Jo Daviess FY07-FY12 vs. LaSalle and Jo Daviess FY19-FY20 (treated as strata) | | Cochran Mantel-Haenszel | | 0.48 | - | 1 | 0.49 | - |
|  | | p-value adjust: Benjamini-Hochberg | | - | - | - | 0.49 | - |
|  | |  | |  |  |  |  |  |
| **Proteotypes (vulnerable vs. less vulnerable) - County** | |  | |  |  |  |  |  |
| LaSalle FY12 vs. Winnebago FY11 | | Fisher’s | | - | - | - | 0.25 | - |
| LaSalle FY12 vs. Jo Daviess CWD area FY07-FY12 | | Fisher’s | | - | - | - | 0.25 | - |
| LaSalle FY19 vs. Jo Daviess CWD area FY20 | | Fisher’s | | - | - | - | 0.08 | - |
| Jo Daviess CWD free FY07-FY12 vs. Jo Daviess CWD area FY07-FY12 | | Fisher’s | | - | - | - | 0.82 | - |
|  | |  | |  |  |  |  |  |
| **Proteotypes (vulnerable vs. less vulnerable) - Temporal** | | | |  |  |  |  |  |
| LaSalle FY12 vs. LaSalle FY19 | | Fisher’s | | - | - | - | 0.89 | - |
| Winnebago FY11 vs. Winnebago FY19 | | Fisher’s | | - | - | - | 0.12 | - |
