# Supplementary material for: County-wide assessments of Illinois white-tailed deer (Odocoileus virginianus) prion protein gene variation using improved primers and potential implications for management
Source: PLoS One. 2022 Nov 30;17(11):e0274640. doi: 10.1371/journal.pone.0274640 (PMC9710747; doi:10.1371/journal.pone.0274640)
Supplement: S3 Table — nd = not detected; past = 2007–2012; JD = Jo Daviess; LS = LaSalle; Win = Winnebago; total unique diplotypes = 67; Diplotypes based on Haley et al. 2021 primers; deer with the haplotypes AA are highly vulnerable to CWD; that deer with a CC or an FF are considered to have a lower vulnerability to CWD; that deer with an AC, AF are less vulnerable than AA alone; Total is the frequency of each diplotype in the entire sample pool; Frequencies are based on Haley primers. (DOCX) [file pone.0274640.s004.docx]

| Diplotype | LS FY 2012 | LS FY 2019 | Win FY 2011 | Win FY 2019 | JD CWD free FY 2007 – FY 2012 | JD CWD free FY 2020 | JD CWD area FY 2007 – FY 2012 | JD CWD area FY 2020 | Total |
| --- | --- | --- | --- | --- | --- | --- | --- | --- | --- |
| AA | 0.030 | 0.050 | 0.090 | 0.080 | 0.104 | 0.104 | 0.167 | 0.104 | 0.081 |
| AB | 0.240 | 0.120 | 0.180 | 0.190 | 0.083 | 0.104 | 0.125 | 0.021 | 0.150 |
| AC | 0.160 | 0.060 | 0.090 | 0.090 | 0.188 | 0.125 | 0.104 | 0.146 | 0.113 |
| AD | nd | 0.050 | 0.070 | 0.030 | 0.021 | 0.021 | 0.021 | nd | 0.030 |
| AE | 0.010 | 0.020 | 0.050 | 0.030 | nd | 0.063 | nd | 0.042 | 0.027 |
| AF | 0.020 | 0.040 | 0.010 | 0.040 | nd | nd | 0.021 | 0.125 | 0.030 |
| AG | 0.010 | nd | 0.030 | 0.010 | nd | nd | nd | 0.021 | 0.010 |
| AH | nd | 0.010 | nd | nd | nd | nd | nd | nd | 0.002 |
| AJ | nd | nd | nd | nd | nd | 0.021 | 0.021 | 0.021 | 0.005 |
| AL | 0.010 | nd | nd | nd | nd | nd | nd | nd | 0.002 |
| ALS-New4 | nd | 0.010 | nd | nd | nd | nd | nd | nd | 0.002 |
| APRNP-odiv33 | nd | nd | nd | nd | 0.021 | nd | nd | nd | 0.002 |
| AU | nd | nd | nd | nd | nd | 0.021 | nd | nd | 0.002 |
| BB | 0.070 | 0.060 | 0.050 | 0.030 | 0.271 | 0.083 | 0.188 | 0.063 | 0.084 |
| BC | 0.100 | 0.140 | 0.090 | 0.110 | nd | 0.042 | 0.083 | 0.167 | 0.098 |
| BD | 0.010 | nd | 0.080 | 0.010 | nd | 0.021 | 0.021 | 0.021 | 0.022 |
| BE | 0.010 | 0.030 | nd | 0.020 | 0.021 | 0.021 | 0.021 | 0.021 | 0.017 |
| BF | 0.020 | nd | 0.010 | 0.020 | 0.021 | 0.063 | nd | 0.042 | 0.019 |
| BG | nd | 0.020 | 0.010 | 0.010 | nd | 0.021 | nd | nd | 0.008 |
| BH | nd | 0.010 | nd | nd | nd | nd | nd | nd | 0.002 |
| BI | 0.010 | 0.020 | nd | nd | nd | nd | nd | nd | 0.005 |
| BJ | nd | nd | 0.010 | nd | nd | 0.042 | nd | 0.021 | 0.007 |
| BU | nd | nd | nd | nd | 0.021 | nd | nd | nd | 0.002 |
| BV | nd | nd | 0.010 | nd | nd | nd | nd | nd | 0.002 |
| CC | 0.080 | 0.040 | 0.080 | 0.070 | 0.083 | 0.042 | nd | 0.042 | 0.059 |
| CD | nd | 0.030 | 0.020 | 0.020 | 0.021 | 0.021 | 0.042 | 0.042 | 0.022 |
| CE | 0.010 | 0.010 | 0.040 | 0.040 | nd | 0.063 | nd | 0.021 | 0.024 |
| CF | 0.010 | 0.020 | nd | 0.020 | nd | 0.021 | 0.042 | nd | 0.014 |
| CG | 0.020 | nd | 0.020 | nd | nd | nd | 0.021 | 0.021 | 0.010 |
| CH | nd | 0.010 | nd | nd | nd | nd | nd | nd | 0.002 |
| CI | 0.010 | nd | nd | nd | nd | nd | nd | nd | 0.002 |
| CJ | nd | nd | 0.010 | nd | 0.021 | nd | 0.021 | nd | 0.005 |
| CL | nd | nd | nd | nd | nd | nd | 0.021 | nd | 0.002 |
| CL203 | nd | 0.010 | nd | nd | nd | nd | nd | nd | 0.002 |
| CLS-New2 | nd | 0.010 | nd | nd | nd | nd | nd | nd | 0.002 |
| CP | nd | nd | nd | 0.010 | nd | nd | nd | 0.021 | 0.003 |
| CPRNP-ovid38 | nd | 0.010 | nd | nd | nd | nd | nd | nd | 0.002 |
| CU | nd | nd | nd | Nd | 0.021 | nd | 0.021 | nd | 0.003 |
| DD | 0.050 | 0.050 | 0.010 | 0.070 | 0.021 | 0.021 | nd | nd | 0.034 |
| DF | 0.010 | 0.050 | 0.010 | 0.010 | nd | nd | 0.021 | nd | 0.015 |
| DG | 0.010 | nd | nd | nd | nd | nd | 0.021 | nd | 0.003 |
| DH | nd | 0.010 | nd | nd | nd | nd | nd | nd | 0.002 |
| DI | nd | nd | nd | 0.010 | nd | nd | nd | nd | 0.002 |
| DJ | nd | nd | 0.010 | nd | nd | nd | nd | nd | 0.002 |
| DLS-New3 | 0.020 | 0.020 | nd | nd | nd | nd | nd | nd | 0.007 |
| DR | 0.050 | nd | nd | nd | 0.021 | 0.021 | nd | nd | 0.012 |
| DV | nd | nd | nd | nd | nd | nd | nd | 0.021 | 0.002 |
| DWin-1 | nd | nd | nd | 0.010 | nd | nd | nd | nd | 0.002 |
| DWin-2 | nd | nd | nd | 0.040 | nd | nd | nd | nd | 0.007 |
| EE | nd | 0.010 | 0.010 | nd | 0.021 | nd | nd | nd | 0.005 |
| EG | nd | 0.020 | nd | nd | nd | 0.021 | nd | nd | 0.005 |
| EP | nd | nd | nd | 0.010 | nd | nd | nd | nd | 0.002 |
| EU | nd | nd | nd | nd | nd | 0.021 | nd | nd | 0.002 |
| FF | nd | nd | nd | 0.010 | nd | nd | nd | nd | 0.002 |
| FG | nd | nd | nd | 0.010 | nd | nd | nd | nd | 0.002 |
| FI | nd | 0.020 | nd | nd | nd | nd | nd | nd | 0.003 |
| FJ | 0.020 | nd | nd | nd | nd | nd | nd | nd | 0.003 |
| FLS-New2 | nd | 0.010 | nd | nd | nd | nd | nd | nd | 0.002 |
| FR | 0.010 | nd | nd | nd | nd | nd | nd | nd | 0.002 |
| GG | nd | 0.010 | nd | nd | nd | nd | nd | nd | 0.002 |
| GPRNP-ovid34 | nd | 0.010 | nd | nd | nd | nd | nd | nd | 0.002 |
| KK | nd | nd | 0.010 | nd | nd | nd | nd | nd | 0.002 |
| LL | nd | nd | nd | nd | nd | nd | nd | 0.021 | 0.002 |
| LP | nd | nd | nd | nd | nd | nd | 0.021 | nd | 0.002 |
| PMimic-L | nd | nd | nd | nd | 0.021 | 0.021 | nd | nd | 0.003 |
| PRNP-ovid33PRNP-ovid33 | nd | 0.010 | nd | nd | nd | nd | nd | nd | 0.002 |
| UPRNP-ovid33 | nd | nd | nd | nd | 0.021 | nd | nd | nd | 0.002 |
| **Total per site** | 25 | 33 | 24 | 26 | 18 | 23 | 19 | 20 | 23.50 |
