# Supplementary material for: County-wide assessments of Illinois white-tailed deer (Odocoileus virginianus) prion protein gene variation using improved primers and potential implications for management
Source: PLoS One. 2022 Nov 30;17(11):e0274640. doi: 10.1371/journal.pone.0274640 (PMC9710747; doi:10.1371/journal.pone.0274640)
Supplement: S4 Table — (DOCX) [file pone.0274640.s005.docx]

| Deer proteotype profile | LS FY12 | LS FY19 | Win FY11 | Win FY19 | JD CWD free FY07 – FY12 | JD CWD free FY 2020 | JD CWD area FY07 – FY12 | JD CWD area FY20 | Overall average proteotype frequency |
| --- | --- | --- | --- | --- | --- | --- | --- | --- | --- |
| AA | 0.510 | 0.490 | 0.600 | 0.490 | 0.583 | 0.563 | 0.563 | 0.333 | 0.519 |
| AC | 0.320 | 0.290 | 0.280 | 0.32 | 0.229 | 0.25 | 0.271 | 0.417 | 0.299 |
| AF | 0.060 | 0.100 | 0.030 | 0.080 | 0.021 | 0.063 | 0.063 | 0.167 | 0.071 |
| AL | 0.010 | nd | nd | nd | 0.021 | nd | nd | nd | 0.003 |
| AU | nd | nd | nd | nd | 0.042 | 0.042 | nd | nd | 0.007 |
| CC | 0.090 | 0.060 | 0.080 | 0.080 | 0.083 | 0.042 | nd | 0.062 | 0.068 |
| CF | 0.010 | 0.030 | nd | 0.020 | nd | 0.021 | 0.042 | nd | 0.015 |
| CL | nd | 0.010 | nd | nd | nd | nd | 0.042 | nd | 0.005 |
| C-L230 | nd | 0.010 | nd | nd | nd | nd | nd | nd | 0.002 |
| CU | nd | nd | nd | nd | 0.021 | nd | 0.021 | nd | 0.003 |
| FF | nd | 0.010 | nd | 0.010 | nd | nd | nd | nd | 0.003 |
| KK | nd | nd | 0.010 | nd | nd | nd | nd | nd | 0.002 |
| LL | nd | nd | nd | nd | nd | 0.021 | nd | 0.021 | 0.003 |
