# Supplementary material for: County-wide assessments of Illinois white-tailed deer (Odocoileus virginianus) prion protein gene variation using improved primers and potential implications for management
Source: PLoS One. 2022 Nov 30;17(11):e0274640. doi: 10.1371/journal.pone.0274640 (PMC9710747; doi:10.1371/journal.pone.0274640)
Supplement: S5 Table — (A) PRNP haplotype, (B) PrP proteoform, and (C) deer susceptibility. Frequencies are calculated by taking the observed frequency based on Haley primers minus the observed frequency based on O’Rourke primers. (DOCX) [file pone.0274640.s006.docx]

| Level of change | LS FY12 | LS FY19 | Win FY11 | Win FY19 | JD CWD free FY07 – FY12 | JD CWD free FY20 | JD CWD area FY07 – FY12 | JD CWD area FY20 | Average change |
| --- | --- | --- | --- | --- | --- | --- | --- | --- | --- |
| Haplotype |  |  |  |  |  |  |  |  |  |
| A | -0.005 | -0.015 | * | -0.005 | * | -0.021 | -0.02 | -0.01 | -0.013 |
| B | -0.015 | -0.01 | -0.015 | -0.005 | * | * | * | -0.01 | -0.011 |
| C | 0.01 | 0.01 | 0.01 | 0.015 | * | 0.031 | * | * | 0.015 |
| D | * | * | 0.005 | -0.005 | * | -0.01 | 0.01 | 0.02 | 0.004 |
| E | * | * | -0.005 | -0.01 | * | * |  | * | -0.008 |
| F | * | -0.005 | * | * | * | * | 0.01 | * | 0.003 |
| G | * | -0.005 | * | * | * | * | * | * | -0.005 |
| I | 0.005 | 0.015 | * | 0.005 | * | * | * | * | 0.008 |
| L | * | * | * | * | * | * | -0.01 | * | -0.010 |
| P | * | * | * | 0.005 | * | * | 0.01 | * | 0.008 |
| PRNP-odvi34 | * | 0.005 | * | * | * | * | * | * | 0.005 |
| PRNP-odvi37 | 0.005 | * | * | * | * | * | * | * | 0.005 |
| LS-New4 | * | 0.005 | * | * | * | * | * | * | 0.005 |
| V | * | * | 0.005 | * | * | * | * | * | 0.005 |
| PrP proteoform | |  |  |  |  |  |  |  |  |
| A | -0.02 | -0.05 | -0.05 | -0.025 | * | -0.03 | -0.02 | * | -0.033 |
| C | 0.015 | 0.05 | 0.05 | 0.025 | * | 0.03 | 0.02 | * | 0.032 |
| F | 0.005 | * | * | * | * | * | 0.01 | * | 0.008 |
| L | * | * | * | * | * | * | -0.01 | * | -0.010 |
| Deer Status |  |  |  |  |  |  |  |  |  |
| Susceptible | -0.05 | -0.09 | -0.02 | -0.05 | * | -0.06 | * | -0.04 | -0.052 |
| less Susceptible | 0.05 | 0.09 | 0.02 | 0.05 | * | 0.06 | 0.02 | 0.04 | 0.047 |
| unknown | * | * | * | * | * | * | -0.02 | * | -0.02 |
